# Micronutrients and Nutrition Status of School-Aged Children in Indonesia

**DOI:** 10.1155/2023/4610038

**Published:** 2023-09-05

**Authors:** Fitrah Ernawati, Efriwati, Nunung Nurjanah, Galih Kusuma Aji, Dwi Hapsari Tjandrarini, Yekti Widodo, Fifi Retiaty, Mutiara Prihatini, Aya Yuriestia Arifin, Dian Sundari, Rika Rachmalina, Salimar, Elisa Diana Julianti, Muhammad Nur Aidi, Ahmad Syauqy

**Affiliations:** ^1^Research Center for Public Health and Nutrition, National Research and Innovation Agency, Cibinong Science Center, Jl. Raya Jakarta-Bogor No. KM 46, Pakansari, Kecamatan Cibinong, Kabupaten Bogor, Jawa Barat 16911, Indonesia; ^2^Research Center for Agroindustry, National Research and Innovation Agency, BJ Habibie Science Center, Setu, Kota Tangerang Selatan, Banten 15314, Indonesia; ^3^Health Policy Agency (BKPK), Ministry of Health, Jl. Percetakan Negara, Jakarta Pusat 10560, Indonesia; ^4^Department of Statistics, IPB University, Jalan Meranti Wing 22 Level 4, Babakan, Dramaga, Kabupaten Bogor, Jawa Barat 16680, Indonesia; ^5^Department of Nutrition Science, Diponegoro University, Jl. Prof Sudarto, Tembalang, Kota Semarang 50275, Indonesia

## Abstract

Micronutrient deficiencies (MNDs) in school-aged children are still a major health problem in Indonesia. This study was designed to examine the status of micronutrients and their relationship to the nutritional status of children aged 5–12 years since an up-to-date database on the micronutrient status of children aged 5–12 years is needed. Data from the 2018 Indonesian Basic Health Research (Riskesdas) were used in this study, with 2456 subjects for analysis. Micronutrient analysis was carried out, including iron status (ferritin, C reactive protein (CRP)), levels of zinc, vitamin D, calcium, and vitamin A (retinol) in school-aged children (5–12 years). The ELISA measurement was applied to measure CRP, ferritin, and vitamin D. Zinc levels were analysed with atomic absorbance spectroscopy (AAS). Moreover, high-performance liquid chromatography (HPLC) was applied to calculate vitamin A. In addition, stunting and thinness data were also obtained from the Riskesdas study. The results showed that the prevalence of stunting and thinness in school-aged children was 11.4% and 9.2%, respectively, showing that the stunting prevalence in the city was lower than in the village (4.5% vs. 6.9%, *P* = 0.000, respectively). In addition, the prevalence of MNDs in Indonesian children was 13.4%, 19.7%, 4.2%, 3%, and 12.7% for ferritin, zinc, calcium, vitamin A, and vitamin D, respectively. The mean serum level of vitamin A and zinc was significantly lower in stunted children compared to normal school children (*P* = 0.010 and *P* = 0.014). The serum concentration of vitamin D was significantly lower in overweight children compared to thin and normal children (*P* = 0.000). Serum values of ferritin, zinc, and vitamin A were significantly higher in overweight children compared to thin and normal children (*P* = 0.000). A poor correlation was observed between the z-score of height-for-age (HAZ) and the levels of zinc (*r* = 0.089, *P* = 0.000), vitamin A (*r* = 0.105, *P* = 0.000), and vitamin D (−0.073, *P* = 0.000). In addition, very weak correlations between z-scores of body mass index-for-age (BAZ) and the serum concentrations of ferritin (0.091, *P* = 0.000), zinc (*r* = 0.115, *P* = 0.000), vitamin A (*r* = 0.137, *P* = 0.000), and vitamin D (*r* = −0.112, *P* = 0.000) were also seen. In conclusion, school-aged children in Indonesia experienced stunting, thinness, and micronutrient deficiency. Furthermore, stunting and thinness were also related to micronutrient deficiencies.

## 1. Introduction

A tiny amount of micronutrients, vitamins, and minerals are consumed, but it is essential to physical and mental growth, specifically for producing enzymes, hormones, and other biomarkers [1, 2]. As of yet, micronutrient deficiencies (MNDs) are still a significant global health problem, and WHO predicts that more than 2 billion people suffer from these deficiencies [1]. School-aged children are one of the at-risk groups to experience MNDs in developing and transition economies countries [3] since micronutrients supplementation is not supplied over again by the government, such as vitamin A. MNDs cause direct and indirect effects on people and society such as deprived health states, inferior scholastic accomplishment, reduced workability, and potency of achievement [4]. In addition, if MNDs happen in childhood, it affects growth and health consequences, including stunting, decreased cognitive and working capacity, repeated infections, growth failure, and increased risk of death [4, 5]. Despite MNDs, children are susceptible to a lack of macronutrients such as stunting, being underweight, and thinness. Thus, relating micro- and macronutrient deficiencies is needed, particularly in Indonesia.

MNDs are reported to be global problems in school-aged children. The studies observed that lack of iron, iodine, folic acid, vitamin A, and zinc is the most commonly reported causes [2, 4, 6]. Anemia is a condition where blood hemoglobin is less than the standard and iron deficiency contributes around 50% [7, 8]. Anemia in school-aged children still becomes a national health problem, showing that the prevalence was more than 20%, contributing 26.4% [9]. In addition, the South East Asian Nutrition Survey (SEANUTS) in 2012 highlighted that the prevalence of iron, vitamin A, and vitamin D deficiency in 5–12 years children was 8.8%, 1.43%, and 44.3%, respectively [10]. Moreover, zinc also happens to be a health problem in Indonesia, but until now, studies regarding its prevalence have not been done nationally. A study in the Bogor district observed that in group-aged children of 11–15 years, zinc deficiency was 38.8% [11]. In addition, a study in Semarang City found that its prevalence was 92% in children between 9 and 12 years old [12]. Other studies in Indonesia reporting the prevalence of calcium deficiency were not found, suggesting that national data must be measured. Furthermore, an update on other MNDs' prevalence is also required since the previous study reported its prevalence in 2012.

Studies outside Indonesia also found similar problems, suggesting that school-aged children risk experiencing MNDs [4, 13–16]. A study in Tanzania found that most rural children had a greater risk of having at least one of the MNDs [13]. In the United States of America (USA), nearly one-third of the children and adult population (>9 years) is at risk of at least one vitamin deficiency or anemia [17]. Moreover, in Ethiopian school-aged children, a study observed that 8 of 10 children had minimally one deficiency of micronutrients, and children with two or greater than two MNDs were 40.5% [18]. Thus, performing MNDs studies becomes essential to examine how big the problem is for Indonesia.

On the other hand, macronutrient problems, including stunting and thinness, are still a national obstacle. At this time, the Indonesian government had put much effort into decreasing this problem, practicing nutrition-sensitive and specific interventions [19]. Stunting prevalence was observed at 30.8% in 2018 [9]. Stunting is a chronic undernutrition status that took place long ago [20]. Thus, it is needed to prevent it before it occurs, meaning that the nutrition approach must be performed when children are in a thinness situation. Literature noted that protein and diversity of micronutrient consumption such as iron, zinc, calcium, vitamin A, and vitamin D contributed to optimal linear growth [21, 22]. Moreover, a study stated that the primary source of micronutrients is obtained from animal sources [23]. However, the food consumption survey results in Indonesia showed that 56% of Indonesian people's food consumption was obtained from cereals. Animal-based products such as meat and poultry, egg, and fish were 2%, 2%, and 5.8%, respectively [24]. A study based on an evaluation of individual food consumption reported that the diets of children (4–10 years) and adolescents (11–18 years) in Indonesia were lower than the required value, showing deficient intakes of iron (26 and 38%, respectively), zinc (60 and 64%, respectively), and vitamin A (57 and 16%, respectively) [25]. School-aged children in Nigeria also experience inadequate intake of micronutrients such as vitamins A, C, B, and Ca [26].

It is imminent, therefore, to assess micronutrient and micronutrient status in the school-age group, considering that data on micronutrient status in this age group are minimal on a national scale.

## 2. Materials and Methods

### 2.1. Study Area and Subjects

A cross-sectional design was applied in this study, which was a part of the 2018 Indonesian Basic Health Research (Riskesdas) study. This study next selected school-aged children between 5 and 12 years. Then, data were coming from 3328 children who had been drawn for serum samples (5–12 years); 841 samples were not used because of insufficient sample volume for analysis (<1000 *µ*L); and 31 samples did not have anthropometric and demographic data. Thus, the final data used for analysis were 2456 subjects, including nutritional status and subjects' characteristics and serum micronutrient values. Serum analysis for micronutrient content such as ferritin, zinc, calcium, vitamin A, and vitamin D was carried out at the Nutrition Laboratory of the Research and Development Center for Biomedical and Basic Health Technology, Ministry of Health Indonesia, from January to December 2021.

### 2.2. Ethical Considerations

The study was reviewed and approved by the National Institute of Health and Research Department (NIHRD) of the Ethical Commission on 26 October 2021 with registered number LB.02.01./2/KE/658/2021. Parents or legal guardians of subjects who participated in the Riskesdas study 2018 signed the informed consent.

### 2.3. Nutritional Assessment

2018 Riskesdas on nutritional assessment was used, performing 2456 data points for analysis. Anthropometric measurements were conducted following standard procedures [27]. Height was measured using a multifunctional height meter with an accuracy of 0.1 cm, and weight was measured using an electronic AND weighing scale (AND, Japan) with an accuracy of 50 grams [28]. Height for age Z-scores (HAZ score) and BMI for age Z-scores (BAZ score) were calculated using the WHO Anthro Plus software. Stunting was defined as having a HAZ score of <−2 SD, whereas normal was organized if the HAZ was between −2 and +1 SD. Thinness, normal weight, and overweight status were classified as having a BAZ score of <−2 SD, −2–+1 SD, and >+1 SD, respectively [29].

### 2.4. Specimen of Serum Collection

The serum samples, archived biological material of the Riskesdas study, were stored in an ultra-low-temperature freezer at 80°C. First, a procedure from the laboratory information management system (LIMS) and Biorepository of Basic Technology Center for Biomedical and Health National Institute of Health and Research and Development was followed to obtain the serum samples. Second, the serums were checked for their number and grouped according to age and serum conditions, whether they were lipemic or not. Last, 2456 serums were used and examined to measure the micronutrient value.

### 2.5. Determination and Classification of Micronutrients Content in Serum

Serum samples from LIMS were prepared at room temperature before analysis, and each sample was checked for its identification number, sex, age group, and condition (whether excellent or lipemic). High-performance liquid chromatography (HPLC) was used to analyse vitamin A. Next, zinc serum was measured using atomic absorbance spectroscopy (AAS). Enzyme-linked immunosorbent assay (ELISA) was then performed to measure vitamin D, C-reactive protein (CRP), and ferritin. Last, an autoanalyzer was used to calculate calcium value.

The ferritin value was adjusted for inflammation if the CRP value was higher than 5, applying a multiplier factor of 0.65 [30]. The ferritin deficiency was categorized if the ferritin value was less than 15 ng/L [8]. Zinc deficiency, second, was classified when the value of zinc serum was less than 0.7 mg/L [31]. Third, a smaller amount of vitamin A (retinol), further down at 20 *µ*g/dL, is considered a vitamin A deficiency [32]. Fourth, putting together the inadequate serum vitamin D level, a concentration of vitamin D (25-hydroxyvitamin D) lower than 30 ng/mL was labeled as vitamin D deficiency [33]. Finally, calcium deficiency was categorized when calcium was less than 8.8 mg/dL [34].

### 2.6. Statistical Analysis

SPSS version 18 was used to analyse the data. First, a one-sample Kolmogorov–Smirnov test was applied to evaluate the data's normality. A descriptive analysis was executed to explain the mean and frequency of each parameter. In addition, a *T*-test was used to observe the difference in micronutrient value in HAZ and BAZ for gender and subjects' residency, as well as the nutrition status of either stunting or normal children. The prevalence of stunting, thinness, and micronutrient status was compared in different subcategories using chi-square analysis. In addition, the ANOVA test was also used to analyse the difference in micronutrient value in thin, normal, and overweight children. Last, to assess the correlation between two continuous variables, Pearson's test was performed, correlating the z scores of HAZ and BAZ with serum values of ferritin, zinc, vitamin A, vitamin D, and calcium. *P* values less than 0.05 were assigned as statistically significant results.

## 3. Results and Discussion

This study used 2456 samples from archived biological material of children aged 5–12 years who were collected in the 2018 Riskesdas study.  Table 1 shows that the majority of respondents lived in urban areas (56.3%), were male (52.7%), and were aged between 5 and 9 years (67.8%).

HAZ and BAZ between boys and girls were not significantly different (−0.54 ± 1.37 and −0.59 ± 1.28, *P* = 0.382 and −0.25 ± 1.65 and −0.22 ± 1.59, respectively, *P* = 0.690) (Table 2). The prevalence of stunting and thinness in children aged 5–12 years was 11.4% and 9.2%, respectively (Table 2). This study also found that the prevalence of stunting in boys and girls was 5.4% and 6%, respectively (*P* = 0.057) (Table 2). In contrast, the prevalence of thinness in boys and girls was 5.3% vs 3.9%, respectively (*P* = 0.140) (Table 2).

Respondents in urban and rural areas were significantly different in nutritional status, showing −0.35 ± 1.36 vs. −0.83 ± 1.24 (*P* = 0.000) and −0.16 ± 1.56 vs. −0.33 ± 1.70 (*P* = 0.009) for HAZ and BAZ, respectively (Table 3). This study observed that stunting prevalence in urban areas was lower than that in rural areas (4.5% vs. 6.9%, *P* = 0.000, respectively) (Table 3). In addition, urban and rural areas corresponded to a similar prevalence of thinness, counting 5% vs. 4.2% (*P* = 0.516), respectively (Table 3).

Table 4 shows the concentrations of serum ferritin, zinc and calcium, vitamin A, and vitamin D based on nutritional status (HAZ and BAZ). The mean serum values of zinc and vitamin A were considerably inferior in stunted rather than normal children (0.82 ± 0.17 and 0.85 ± 0.19 mg/L, *P* = 0.014 and 1.08 ± 0.24 and 1.13 ± 0.25 *µ*mol/L, *P* = 0.010, respectively). However, serum concentrations of ferritin, calcium, and vitamin D were no different between stunted and normal nutritional status. A higher value of ferritin was observed in overweight (52.63 ± 37.13 ng/mL) than in thinness (42.33 ± 40.83 ng/mL) and normal (43.29 ± 33.03 ng/mL) (*P* = 0.000). Serum zinc, furthermore, was significantly higher in overweight (0.90 ± 0.19 mg/L) compared to thinness (0.82 ± 0.20 mg/L) and normal (0.83 ± 0.18 mg/L) (*P* = 0.000). This study also found that the concentration of vitamin A was significantly higher in overweight children compared to thin and normal children (1.19 ± 0.23, 1.09 ± 0.28, and 1.11 ± 0.24 *µ*mol/L, respectively, *P* = 0.000). Moreover, overweight children experienced lower serum concentrations of vitamin D in contrast with thin and normal children (41.67 ± 17.92, 47.19 ± 16.87, and 46.95 ± 18.53 ng/dL, respectively, *P* = 0.000). In contrast, serum calcium was not different between thin, normal, and overweight children (*P* = 0.892).

MNDs prevalence was depicted in Table 5. In this study, 13.4% of respondents had ferritin deficiency; dividing by gender showed that the prevalence for boys and girls was 7.2% and 6.2% (*P* = 0.747), respectively. Zinc deficiency occurred in 19.7% of the school children, showing a prevalence of 11.2% and 8.5% (*P* = 0.052) for boys and girls, respectively. This study also observed that deficiency of calcium, vitamin A, and vitamin D was 4.2% and 3%, and 12.7%, respectively. In addition, the deficiency of calcium and vitamin A was significantly lower in girls compared to boys (1.7% vs 2.4%, *P* = 0.000 and 1.1% vs 1.8%, *P* = 0.015, respectively). In contrast, girls had a higher prevalence than boys of vitamin D deficiency (8.3% vs 4.4%, *P* = 0.000, respectively).

Pearson's correlation analysis resulted in significant outputs between z-scores of HAZ and the levels of zinc (*r* = 0.089, *P* = 0.000), vitamin A (*r* = 0.105, *P* = 0.000), and vitamin D (*r* = −0.073, *P* = 0.000) (Table 6). A very weak positive correlation was observed between BAZ and the values of ferritin (0.091, *P* = 0.000), zinc (*r* = 0.115, *P* = 0.000), and vitamin A (*r* = 0.137, *P* = 0.000) (Table 6). In addition, vitamin D (*r* = −0.112, *P* = 0.000) was negatively correlated with BAZ, showing a poor magnitude (Table 6).

## 4. Discussion

This study revealed an overview of the present data on the nutritional status of Indonesian children aged 5–12 years. The available data showed that stunting and thinness prevalence (11.4% and 9.2%, respectively) are persistent problems in Indonesian school-aged children [35]. This result is similar to the stunting prevalence of children aged 6–12 years in Ethiopia (11.6%) and much lower than in Africa (22%), South-East Asia (29%), Latin America (16%), the Eastern Mediterranean (24%), and the Western Pacific (28%) [3, 36]. Regarding thinness, this study showed a higher prevalence than in Latin America (6%) and much lower than in Ethiopia (10.8%), Africa (36%), South-East Asia (34%), Eastern Mediterranean (13%), and Western Pacific (14%) [3, 36]. This achievement in stunting and thinness prevalence might be explained by the National Movement for Stunting Prevention and Multi-Sector Partnership Cooperation in Indonesia, which includes specific and sensitive, nutrition interventions for children in their first 1,000 days of life in various development activities beyond the health sector targeting the general population [19]. In addition, the Indonesian government's commitment and ambition to address stunting have also contributed to this achievement [37]. However, the prevalence of both stunting and thinness in Indonesian children aged 5–12 years was still in the medium category [35]. Nutrition interventions aimed at improving nutritional status, such as promoting dietary diversity and balanced diets and community-based and school-based approaches to micronutrient supplementation (especially iron), have shown diverse results and are not yet fully effective due to Indonesia's vast territory. Similar efforts to improve nutrition in school-aged children have also been undertaken in Ethiopia [36], resulting in a similar prevalence of stunting in Indonesia and Ethiopia of 11.4% and 11.6%, respectively. This study also could not detect the mean difference between HAZ and BAZ between boys and girls. However, a study in low- and middle-income countries (LMICs) conducted on children and adolescents suggested that low nutritional status (stunting and thinness) was higher in boys compared to girls [38].

The mean difference between HAZ and BAZ scores among schoolchildren was significantly superior in the city rather than in rural. A study in LMICs confirmed this finding that living in urban areas, having higher socioeconomic status, safer food security, and healthier eating habits such as breakfast tradition and healthy snacks consumption reduced the chance of stunting and thinness in school-aged children and adolescents [38]. Undernutrition among this age group is often neglected even though a study in rural Indian boys aged ≥5 years showed that prepubertal height deficits among stunted children were carried into adult height, increasing the risk of potentially irreversible loss of growth and cognitive functions [39]. Therefore, this problem should be addressed earlier, and intervention must provide sufficient time to evaluate the growth of this age group. Screening for low HAZ and BAZ scores in children through school or pediatric primary health care should be considered a national program priority.

In addition to nutritional status, this current study also showed the prevalence of MNDs on a national scale. The results observed that in school-aged children, the prevalence of ferritin, zinc, calcium, vitamin A, and vitamin D was 13.4%, 19.7%, 4.2%, 3%, and 12.7%, respectively. The national prevalence of zinc and calcium deficiency in school-aged children was reported for the first time in this study. In addition, this finding also updated the prevalence data from 2012 for iron, vitamin A, and vitamin D deficiency in 5 to 12-year-old children, which was 8.8%, 1.43%, and 44.3%, respectively [10]. This study showed that the highest micronutrient deficiency proportion was found in ferritin and zinc. A study on Ethiopian children aged 10–14 confirmed this study [16]. A national food consumption survey of Indonesian school-aged children (5–12 years) might explain this result, reporting less than half of children consumed iron- and zinc-source foods, for example, meat (24.6%), fish (25.1%), eggs (43.6%), and soybeans (35.9%) [24]. In addition, this study found that micronutrient deficiency was more prevalent in girls than boys. A study on the US population supported this finding that points to the risk of vitamin deficiency or anemia being more frequent in girls than in boys [17].

Ferritin serum levels were lower in thin and normal school-aged children than in those who were overweight (*P* = 0.000). A study among Saudi children and adolescents confirmed that thin children were likelier to have lower serum ferritin than normal BMI children [40]. Iron deficiency indicated by low serum ferritin impairs education achievement, productivity, and ability, and chronic anemia damages growth and the function of the cardiovascular system [41, 42].

Zinc serum level was lower in thin and stunted school-aged children compared with their counterparts in this study. This result is in accordance with the study of Indian children and adolescents, which concluded a higher number of low serum zinc levels in underweight and stunted children [43]. Evidence has shown that zinc significantly affects children's linear growth and weight gain [44–46]. However, a meta-analysis of randomized controlled trial studies in children aged 1–8 years found that zinc supplementation did not significantly affect weight gain or the scores of weight-for-age, length/height-for-age, and weight-for-height [47].

This study found that stunted and thin school-aged children had lower vitamin A serum levels than the standard group. Vitamin A's role in controlling average growth and development through its direct effect on growth hormone and its carrier protein may explain this result [48, 49]. Moreover, a study in Uganda of under-five children supported this study's results that concluded vitamin A deficiency was independently connected to higher odds of stunting growth (adjusted odds ratio, 1.43 (95% CI: 1.08–1.89), *P* = 0.01) [49].

Vitamin D has significant contributions to a child's growth. Evidence from child and adolescent studies showed that declining linear growth was related to vitamin D deficiency [50–52]. Vitamin D contributes to bone mineralization, increases the sensitivity of the growth plate cells to growth hormone, and promotes the growth plate cells to appropriately differentiate and mature [50, 53]. This study found that overweight school-aged children suffered lower vitamin D serum values than thin and normal children (*P* = 0.000). The reviewed study stated that overweight and obese people tend to have vitamin D deficiency, ranging from 5% to 90% [54, 55]. A potential explanation of this phenomenon is that overweight and obese people experience inflammation of adipocyte tissues [54] and vitamin D dilution due to a higher distributed volume of serum [55]. In addition to calcemic functions, it is also found that vitamin D plays a prominent role in energy metabolism, in particular glucose and fat metabolism in adipocyte cells that affects insulin sensitivity [54]. Thus, overweight and obese children with vitamin D deficiency are needed to increase vitamin D consumption before adulthood, helping to reduce the risk of metabolic syndromes such as type 2 diabetes mellitus, dyslipidemia, hypertension, and others.

Further analysis showed that zinc and vitamin A exhibited a very weak positive correlation with the HAZ and BAZ scores. On the other hand, vitamin D negatively correlated with both HAZ and BAZ in a very weak capacity. In addition, ferritin was only poorly correlated with BAZ. All of this evidence reported that undernutrition in school-aged children was related to low serum levels of several vitamins and minerals. Reports elucidating the correlation between HAZ, BAZ, and micronutrient value were limited. A study in Cameroon children found that HAZ was significantly correlated to zinc, calcium, and iron with a poor magnitude, providing coefficient values of 0.093, 0.029, and 0.111, respectively [56]. In addition, in preschool children of Sri Lanka, it was reported that vitamin D was negatively correlated with WAZ (weight for age) and WHZ (weight for height) [57]. These consistent findings may be due to similar energy intake among this group. Indonesian children consumed energy of 1.636 kcal whereas Cameroon and Sri Lanka children consumed energies of 1.640 kcal and 1.708 kcal, respectively [58, 59]. Thus, to tackle the MNDs, it is important to provide nutritious foods to children. One of the best approaches is through the education system, where schooling may serve as an effective entry point for implementing nutrition intervention programs that can efficiently reach children. These programs could include nutritious meals at school with a particular focus on food sources rich in iron and zinc, as well as vitamin and micronutrient supplementation, deworming prophylaxis, and nutrition education emphasizing balanced nutrition. These programs also should be monitored and regularly evaluated. In addition, it is essential to encourage children to have breakfast before starting their day and provide sufficient time to eat lunch at school. Having ≥20 minutes of lunch is related to considerable consumption of grains, meats, milk, and vegetables [60]. All of the approaches might be effective in boosting the intake of micronutrients and addressing deficiencies.

The strength of this study was the nationally representative data from the Indonesian Ministry of Health survey with a large sample size. Moreover, data from biochemical analyses of ferritin, zinc, calcium, vitamin A, and vitamin D provide good indicators of MNDs among school-aged children. However, the use of cross-sectional data was a limitation of this study which does not allow for causal inferences.

## 5. Conclusions

The current study observed that the prevalence of MNDs for ferritin, zinc, calcium, vitamin A, and vitamin D in Indonesian school-aged children was 13.4%, 19.7%, 4.2%, 3%, and 12.7%, respectively. In addition, stunting and thinness prevalence were 11.4% and 9.2%, respectively. Moreover, chronic nutritional status was correlated with zinc and vitamin A. Furthermore, acute nutritional status (BAZ) was correlated with a lack of micronutrients, specifically ferritin, zinc, and vitamin A. These findings indicate that micronutrient deficiencies in school-aged children must be addressed immediately by evaluating and planning health programs to address these problems, such as supplementation and fortification of micronutrients, deworming, vector control, nutrition and health education, sanitation improvement, clean water supply, and other appropriate approaches.

## Figures and Tables

**Table 1:** Demographic characteristics of children aged 5–12 years.

Variables	*N*	(%)
*Residences*		
Urban	1382	56.3
Rural	1074	43.7

*Sex*		
Boys	1294	52.7
Girls	1162	47.3

*Age group*		
5–9 years	1664	67.8
10–12 years	792	32.2

**Table 2:** The mean score of HAZ and BAZ and nutritional status prevalence of Indonesian children aged 5–12 years regarding gender.

Variables	*N*	Mean ± SD	95% CI		*P* value		*P*
			Upper	Lower			
*HAZ*						*N (%), stunting*	
Boys	1294	−0.54 ± 1.37	−0.61	−0.46	0.382	132 (5.4%)	0.057
Girls	1162	−0.59 ± 1.28	−0.66	−0.51		147 (6%)	
Total	2456	−0.56 ± 1.33	−0.61	−0.51		279 (11.4%)	

*BAZ*						*N (%), thinness*	
Boys	1294	−0.25 ± 1.65	−0.34	−0.16	0.690	129 (5.3%)	0.140
Girls	1162	−0.22 ± 1.59	−0.31	−0.13		96 (3.9%)	
Total	2456	−0.24 ± −1.62	−0.30	−0.17		225 (9.2%)	

HAZ, height-for-age Z-score; BAZ, BMI-for-age Z-score.

**Table 3:** The mean score of HAZ and BAZ and nutritional status prevalence based on the residency of children 5–12 years in Indonesia.

Variables	*N*	Mean ± SD	95% CI		*P*		*P*
			Upper	Lower			
*HAZ*						*N (%), stunting*
Urban	1382	−0.35 ± 1.36	−0.42	−0.28	0.000^*∗∗*^	111 (4.5%)	0.000
Rural	1074	−0.83 ± 1.24	−0.91	−0.76		169 (6.9%)
Total	2456	−0.56 ± 1.33	−0.61	−0.51		280 (11.4%)

*BAZ*						*N (%), thinness*
Urban	1382	−0.16 ± 1.56	−0.24	−0.08	0.009^*∗∗*^	122 (5%)	0.516
Rural	1074	−0.33 ± 1.70	−0.44	−0.23		103 (4.2%)
Total	2456	−0.24 ± −1.62	−0.30	−0.17		225 (9.2%)

HAZ, height-for-age Z-score; BAZ, BMI-for-age Z-score. ^*∗∗*^Indicates a significant value of <0.01.

**Table 4:** Level of micronutrients (mean ± SD) according to nutritional status in children aged 5–12 years.

Nutrition status	Ferritin (ng/mL)	*P*	Zinc (mg/L)	*P*	Calcium (mg/dL)	*P*	Vit A	*P*	Vit D (ng/dL)	*P*
	mean ± SD		mean ± SD		mean ± SD		mean ± SD		mean ± SD	
*HAZ*										
Stunting (*n* = 279)	44.57 ± 29.25	0.872	0.82 ± 0.17	0.014^*∗*^	10.50 ± 0.72	0.840	1.08 ± 0.24	0.010^*∗*^	47.28 ± 14.73	0.218
Normal (*n* = 2177)	44.94 ± 35.41		0.85 ± 0.19		10.56 ± 4.83		1.13 ± 0.25		45.85 ± 18.80	
Total (*n* = 2456)	44.90 ± 34.76		0.84 ± 0.19		10.55 ± 4.55		1.12 ± 0.25		46.01 ± 18.38	

*BAZ*										
Thinness (*n* = 225)	42.33 ± 40.83^b^	0.000^*∗∗*^	0.82 ± 0.20^b^	0.000^*∗∗*^	10.44 ± 1.08	0.892	1.09 ± 0.28^b^	0.000^*∗∗*^	47.19 ± 16.87^a^	0.000^*∗∗*^
Normal (*n* = 1785)	43.29 ± 33.03^b^		0.83 ± 0.18^b^		10.55 ± 5.31		1.11 ± 0.24^b^		46.95 ± 18.53^a^	
Overweight (*n* = 447)	52.63 ± 37.13^a^		0.90 ± 0.19^a^		10.62 ± 0.91		1.19 ± 0.23^a^		41.67 ± 17.92^b^	
Total (*n* = 2456)	44.90 ± 34.76		0.84 ± 0.19		10.55 ± 4.55		1.12 ± 0.25		46.01 ± 18.38	

HAZ, height-for-age Z-score; BAZ, BMI-for-age Z-score. ^*∗*^indicates a significant value of <0.05 and ^*∗∗*^indicates a significant value of <0.01.

**Table 5:** Values of micronutrients (mean ± SD) and deficiency proportion (%) characterized by sex in children aged 5–12 years.

Micronutrients	Mean ± SD (range)		Deficiency (%)		*P*	Sexes Combined, *N* = 2456
	Boys, *N* = 1294	Girls, *N* = 1162	Boys, *N* (%)	Girls, *N* (%)		Deficiency, *N* (%)
Ferritin (ng/mL)	49.11 ± 42.34 (46.80–51.42)	40.21 ± 22.73 (38.90–41.51)	176 (7.2%)	153 (6.2%)	0.747	329 (13.4%)
Zn (mg/L)	0.84 ± 0.19 (0.83–0.86)	0.84 ± 0.19 (0.83–0.85)	274 (11.2%)	210 (8.5%)	0.052	484 (19.7%)
Calcium (mg/dL)	10.50 ± 1.05 (10.45–10.57)	10.59 ± 6.53 (10.22–10.97)	60 (2.4%)	42 (1.7%)	0.000^*∗∗*^	102 (4.2%)
Vit A (*µ*mol/L)	1.11 ± 0.25 (1.09–1.12)	1.13 ± 0.24 (1.12–1.15)	45 (1.8%)	28 (1.1%)	0.015^*∗*^	73 (3.0%)
Vit D (ng/mL)	48.16 ± 19.37 (47.10–49.22)	43.62 ± 16.90 (42.64–44.59)	107 (4.4%)	204 (8.3%)	0.000^*∗∗*^	311 (12.7%)

^*∗*^The *P* value indicated a significant prevalence deficiency of <0.05. ^*∗∗*^The *P* value indicated a significant prevalence deficiency of <0.01.

**Table 6:** Correlation between micronutrient value and nutritional status (HAZ and BAZ) of 5- to 12 -year-old children.

Micronutrients	HAZ	BAZ
Ferritin (ng/mL)	−0.003 (*P* = 0.870)	0.091^*∗∗*^ (*P* = 0.000)
Zn (mg/L)	0.089^*∗∗*^ (*P* = 0.000)	0.115^*∗∗*^ (*P* = 0.000)
Calcium (mg/dL)	−0.006 (*P* = 0.770)	0.013 (*P* = 0.519)
Vit A (*µ*mol/L)	0.105^*∗∗*^ (*P* = 0.000)	0.137^*∗∗*^ (*P* = 0.000)
Vit D (ng/ml)	−0.073^*∗∗*^ (*P* = 0.000)	−0.112^*∗∗*^ (*P* = 0.000)

HAZ, height-for-age Z-score; BAZ, BMI-for-age Z-score. ^*∗*^Significant value of <0.05; ^*∗∗*^significant value of <0.01.

## Data Availability

The data used to support the findings of this study have not been made available due to the Indonesian Ministry of Health policy.
